# Spectral Imaging Analysis for Ultrasensitive Biomolecular Detection Using Gold-Capped Nanowire Arrays

**DOI:** 10.3390/s18072181

**Published:** 2018-07-06

**Authors:** Yi-Hsin Tai, Po-Han Fu, Kuang-Li Lee, Pei-Kuen Wei

**Affiliations:** 1 Research Center for Applied Sciences, Academia Sinica, Taipei 11529, Taiwan; yhtai@gate.sinica.edu.tw (Y.-H.T.); pohan521@gmail.com (P.-H.F.); kllee@gate.sinica.edu.tw (K.-L.L.); 2 Institute of Biophotonics, National Yang-Ming University, Taipei 11221, Taiwan; 3 Institute of Optoelectronic Sciences, National Taiwan Ocean University, Keelung 20224, Taiwan

**Keywords:** surface plasmon resonance, nanostructures, multiple-wells detection, spectral integration, threshold analysis

## Abstract

A spectral integration combined with a threshold method for the analysis of spectral scanning surface plasmon resonance (SPR) images can significantly increase signal recognition at low concentration of antibody solution. The 12-well SPR sensing plates consisted of gold-capped nanowire arrays with 500-nm period, 80-nm linewidth and 50-nm gold thickness which were used for generating multiple SPR images. A threshold method is introduced to eliminate background noises in spectral scanning images. Combining spectral integration and the threshold method, the detection limit of antibody concentration was 1.23 ng/mL. Using multiple-well SPR sensing plates and the proposed analytical method, multiple kinetic responses with spectral and spatial information on different sensing areas can be sensitively measured.

## 1. Introduction

Surface plasmon resonance (SPR) imaging is a method for kinetic studies of bio-interactions with label-free and high-throughput advantages [1,2,3,4,5,6]. Compared to traditional SPR sensing techniques, the SPR imaging method records more information from the sensing area. The spectral response on the SPR sensing surface is usually obtained by a spectrometer. By contrast with interference signals from SPR phase detection [7,8,9], the detected signal is from the shift of the specific wavelength in the spectrum. Using hyper-spectrometer technology [10,11], the spectral responses become two-dimensional in space which can be directly mapped on to the captured image. The SPR imaging method is capable of measuring surface bio-interactions with spatial distribution, which is useful for measuring multiple protein–protein interactions or DNA hybridizations [12,13,14]. However, due to Brownian motion [15,16] and the effect of fluidics [17,18], the surface immobilization distribution is usually non-uniform especially at low concentration. Therefore, a distinguishable signal change in the SPR image at low concentration is required to enhance the detection limit [19,20]. In this work, the spatial signals for increasing the surface sensitivity at low protein concentrations were enhanced by a threshold method. The multiple SPR sensing plates composed of gold-capped nanowire arrays were fabricated by the nanoimprinting method and thermal evaporation [21]. Combined with a fluidic cover, a sensing plate consisting of 12 SPR wells was measured with different protein concentrations simultaneously. The SPR images were analysed based on the spectral integration analysis (SIA) method for spectral signals and the threshold method for spatial information. By choosing the threshold that equals the average plus one standard deviation of the control SPR image, the detection limit for anti-bovine serum albumin antibody (anti-BSA) is significantly improved up to two orders of magnitude without using extra signal enhancement [22,23,24] or protein concentrated system [25,26,27].

## 2. Experimental Section

### 2.1. Chip Fabrication

**Figure 1:**
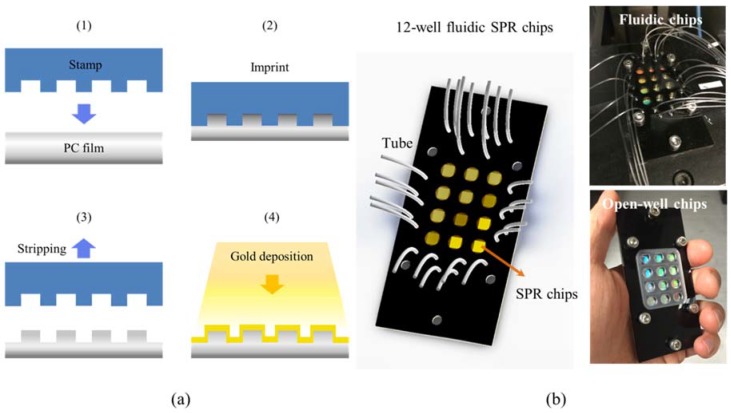
(**a**) The 12-well surface plasmon resonance (SPR) sensing plates were fabricated by nanoimprinting 12 nanowire arrays on a plastic film. The nanowire array had a 500-nm period and 80-nm linewidth and was coated with 50-nm gold film; (**b**) the 12-well SPR sensors were sealed by a fluidic cover for the injection of different medium.

Figure 1a shows the fabrication process of multiple-well SPR chips. By using nanoimprint technology [28], nanowire arrays with 500-nm period and 80-nm linewidth were imprinted on a polycarbonate (PC) plastic film. The PC film shaped with nanowire arrays was deposited with 50-nm gold film for inducing Fano-like SPR resonance [21,29]. The Fano resonance results from the coupling of the cavity mode, which was created by the localized surface plasmon resonance (LSPR), and Bloch wave surface plasmon polariton (BW-SPP) for detecting the protein immobilization with high sensitivity. The performance of the sensor has been discussed in our previous work [21]. The figure of merit (FOM) of the gold nanowire array was about 166, and the detection limit of intensity was about 3 × 10^−5^ RIU. If 0.2% intensity stability can be achieved, the detection limit is 4 × 10^−6^ RIU, which is close to the prism-based SPR sensor using angular interrogation, but is one order of magnitude smaller than the phase SPR sensor, which is about 5 × 10^−7^ RIU [7]. Next, the gold-capped nanowire arrays were combined with a 12-well fluidic cover as shown in Figure 1b. Each well was independently injected with medium without interfering with others. In the SPR image measurement, the transmission spectra of the 12-well SPR fluidic chip was read by a spectrometer for kinetic bio-interaction measurements.

### 2.2. Optical Setup

**Figure 2:**
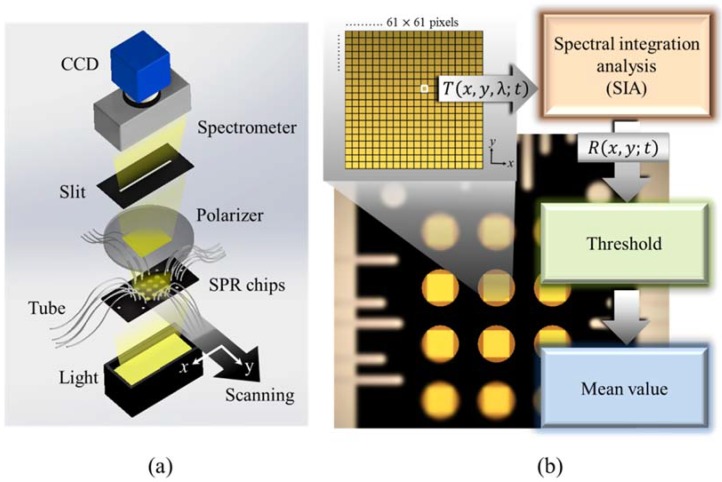
(**a**) The spectral SPR scanning system for capturing multiple SPR spectral images from a 12-well SPR fluidic chip. (**b**) The effective detection area contained 61 × 61 pixels. The transmission spectra of each pixel were recorded for further analysis based on the SIA.

The spectral scanning SPR image system is shown in Figure 2a. A light source, which was put below the chip, emitted white light and passed the chip from the bottom to the top. The optical SPR signals on the capped gold nanowire arrays were filtrated by a linear polarizer and a slit. Then, the transmission light with SPR signals was read by a spectrometer and recorded as an SPR image by a charge-coupled device (CCD). We obtained the complete spectrum image I(x,y,λ) for each pixel by the two-dimensional CCD, which represents a full slit spectrum (x,λ). The spectral imaging device for spatial scanning obtains slit spectra by projecting a strip of the SPR sensing chip onto a slit and dispersing the slit image with a grating. With a line-scan system, the spatial dimension is collected through platform scanning along the y-direction. The pixel size on the SPR image was 52 μm^2^/pixel. According to the size of SPR sensing area imaged on the CCD, a 61 × 61-pixels SPR image was chosen to be the effective detection area for each well. Each pixel in the detection area was set as a unit and had its spectral information. To avoid the SPR signal changes from the change of bulk refractive index of the medium, the SPR image was scanned and recorded under a stable refractive index environment by flowing deionized (DI) water. In the protein interaction experiments, the DI water environment was recorded as the initial SPR image. Next, serum albumin antibody (BSA) 1 mg/mL in DI water was injected into the wells at a flow rate of 3 μL/min for 2 h. After flowing BSA solution, the 12 sensing wells were washed by DI water at a flow rate of 300 μL/min for 10 min. Next, different anti-BSA concentrations of 1 ng/mL, 10 ng/mL, 100 ng/mL, 1 μg/mL and 10 μg/mL in DI water were injected into different well at a flow rate of 300 μg/min for 3 h. Finally, DI water with a 300-μL/min flow rate was injected for cleaning non-specific binding for 10 min. Figure 2b shows the schematic for the SPR spectral analysis, which was carried out by our in-house developed program. Each chip is a square with a side length of 5.00 mm and the effective detection area on a chip is inside a square with a side length of 3.17 mm while the responses outside the square are neglected. To present digitalized images for the visualization of response distribution, every effective detection area was meshed into 61 × 61 grids (pixels). Thus, the area of each pixel is 52 μm^2^, which is large enough to avoid being influenced by adjacent pixels so that the visualization is sufficiently accurate to present the result. The responses for the SPR signals were obtained by first using the SIA method to deal with the spectral information and then the spatial information was filtered using a threshold method. Finally, the responses for pixels were summed and their mean value was recoded. Using the above algorithm, the detection limit can be improved by about two orders of magnitude as compared to conventional peak wavelength measurement using a spectrometer.

## 3. Result and Discussion

### 3.1. Spectral Integration Analysis (SIA)

**Figure 3:**
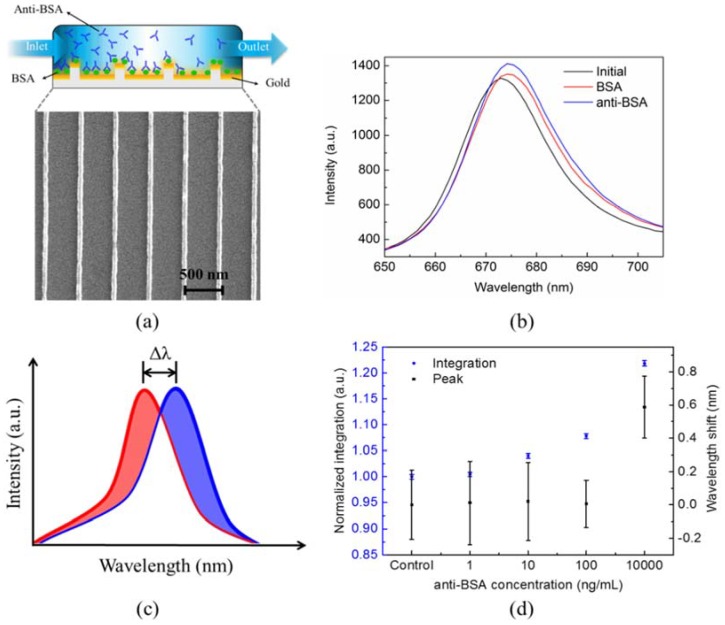
(**a**) Bovine serum albumin (BSA) and anti-BSA were sequentially immobilized on the capped gold nanowire array. (**b**) Each pixel has its own spectral response. Transmission spectra of each pixel under different medium conditions were recorded for further analysis. (**c**) The illustration for peak-wavelength shift and spectral integration analysis for calculating the SPR responses during the protein–protein interactions. (**d**) The calculated peak-wavelength shift and spectral integration for BSA protein interacted with different concentrations of anti-BSA.

Figure 3a shows the illustration of the BSA and anti-BSA specific binding on gold-capped nanowire arrays, and the scanning electron microscope image shows the imprinted nanowire arrays. Figure 3b shows the transmission spectra with DI water only, BSA immobilization, and anti-BSA binding on a single pixel area. In the SPR measurement, the usual way is to measure the peak wavelength shift or intensity change near the SPR peak. Figure 3c shows the schematic of the peak shift analysis method. The peak shift equals the change of the peak wavelength of the transmission spectra owing to protein immobilization on the sensing surface. On the other hand, as shown in Figure 3c, by considering both the peak wavelength shifts and intensity changes, the SIA method is introduced to determine the response. The SIA has been conducted for the analysis of SPR spectrum for various metallic nanostructures, including nanohole SPR sensors and nanogrid array sensors [30,31]. It can significantly improve the signal-to-noise ratio and thus enhance the detection limit. For the SIA calculation, each pixel requires initial transmission spectra and transmission spectra at different time, namely, $T_{i}\;(x,y,\lambda ;t=0)$ and $T_{f}\;(x,y,\lambda ;t)$. The integrated response R(x,y) based on the SIA is the summation of the absolute values of the differences between $T_{i}\;(x,y,\lambda )$ and $T_{f}\;(x,y,\lambda )$ for $\lambda =\lambda_{1}\sim \lambda_{2}$, which is expressed as:

(1) $$\;R(x,y)\;=\;\overset{\lambda_{2}}{\underset{\lambda =\lambda_{1}}{\sum }}\left|T_{i}\;\left(x,y,\lambda \right)-T_{f}\;(x,y,\lambda )\right|$$

Here, we take *λ*_1_ = 600 nm and *λ*_2_ = 800 nm. Figure 3d shows the result using the peak shift analysis and SIA under different anti-BSA concentrations. The spectrum of each pixel was calculated using the SIA method and peak-wavelength shift. Figure 3d shows the results from the all sensor pixels for various concentrations of anti-BSA. The standard deviations (STDs) for the SIA signals were obtained from the measured results at different times. The variation of SIA signals was 0.4%. For the wavelength shift measurement, the resolution of the spectrometer was about 0.4 nm. Based on the STD, the limit of detection (I_LOD_) was defined by I_LOD_ = I_control_ + 3 × STD. I_control_ and STD were the DI water signal and its standard deviation. In Figure 3d, the wavelength shift measurement with 0.4 nm resolution can only detect ~1 μg/mL antibody. Using the SIA method, the calculated LOD of anti-BSA was improved to 2.13 ng/mL.

**Figure 4:**
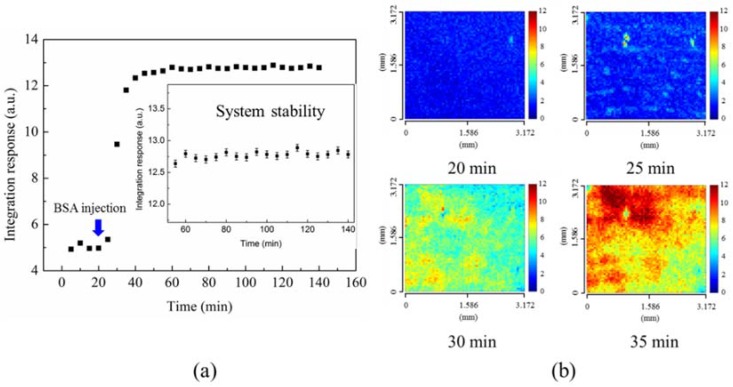
(**a**) 1-mg/mL BSA solution was injected to the sensing area after 20 min. The time-lapse mean value was determined from the SPR scanning image. The STD was obtained from the measured results at different times. (**b**) BSA immobilization on the sensing surface at different times. The time-evolution 2D SPR images in the detection area showed the non-uniform distribution.

Figure 4a shows the time-lapse plot for the immobilization of BSA molecules on the gold surface. It shows the average results from all the sensor pixels for different interaction times. The variations between pixels can be observed in Figure 4b. The time-lapse mean value of R(x,y;t), denoted as M(t), was calculated by ${M(t)\;=\;\Sigma }_{j=1\;}^{61}\Sigma_{i=1\;}^{61}\;R(x_{i},y_{i}{;t)/61}^{2}$. The timer begins when the deionized water is injected into the fluidic channel, and the colormaps of R(x,y) distribution for *t* = 50–150 min are displayed in sequence in the Supplementary Movie S1. The stability of signals before anti-BSA and BSA interactions can be seen in the time-lapse plot. The variation of signals is 0.43%. When the BSA solution is injected at *t* = 20 min, the BSA adhesion on the gold surface begins. Figure 4b shows the maximum change of kinetic SPR images for 25–35 min after the timer begins. It is the time interval when the BSA solution was continuously flowed into the chamber. In the beginning of BSA adsorption, the analytes tend to form several aggregations rather than a thin, uniform BSA layer. After two hours, sufficient BSA are immobilized on the gold surface and the response reaches the steady state. Note that the non-uniform distribution still exists. The colormaps, histograms, and mean value M of R(x,y) with error bars were calculated using the SIA for three-hour static adsorption under different anti-BSA concentration injections from 1 ng to 10 μg/mL The spectrum of each pixel was calculated using the SIA method. The SIA signals were quite uniform in the beginning. When BSA immobilized on the surface, the deviation between pixels increased with time.

### 3.2. Threshold Method

**Figure 5:**
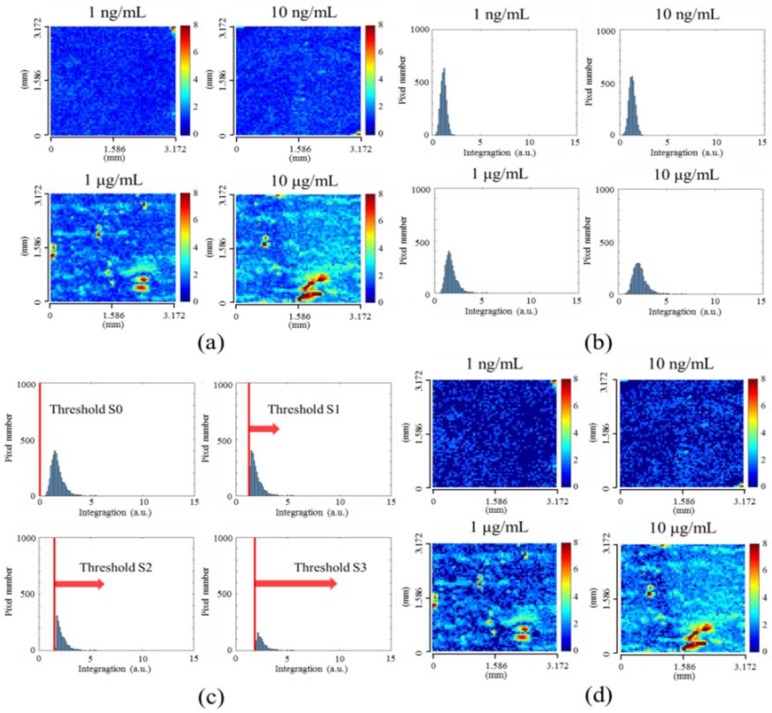
Anti-BSA concentration with 1 ng, 10 ng, 100 ng, 1 μg and 10 μg/mL flowed after BSA immobilization. (**a**) The SPR image showed the distribution in the effective area. (**b**) The histograms of R(x,y) were calculated by spectral integration analysis (SIA) at different anti-BSA concentration. (**c**) The modified histograms with different threshold values, with the average plus 0(S0), 1(S1), 2(S2), and 3(S3) times standard deviations of the control experiments. The x-axis and y-axis shown in (**b**,**c**) are the integration response R and the number of pixels. (**d**) By eliminating the background noise, the contrast of SPR images become more pronounced at low anti-BSA concentration, respectively.

Figure 5 show the R(x,y) images for different anti-BSA concentration. Similar to the result of dynamic measurement for BSA injection, the analytes are not sufficient to form a single layer but several aggregations for low anti-BSA concentrations. As anti-BSA concentration increases up to 1 μg/mL, most of the pixels are deposited with at least one layer of the analytes, and the non-uniform distribution still exists. Figure 5b shows statistic distribution of R(x,y) values for various concentrations. There is a small deviation of the distribution when the concentration is low. When the concentration increases, the deviation increases. This verifies the non-uniform interactions between BSA and anti-BSA proteins. From the mean values M of the distribution, the detection limit is around 10 ng/mL. For low concentrations below 10 ng/mL, the difference of M remains obscured because the signal may be mixed up with unwanted noises by averaging the non-uniform responses. Based on the visualized distribution images in Figure 5a, it is reasonable to deduce that the detection limit can be improved by an analysis method which is effective in eliminating unwanted noises especially for low concentrations. By simply adding a discriminant based on the SIA, a threshold value T is introduced and the pixels for R(x,y)<T are considered as noises. The newly built equation is expressed as $R^{\prime }(x,y)\;=\;[R(x,y)>T]\times R(x,y)$, where $R^{\prime }(x,y)=R(x,y)$ if R(x,y)>T, otherwise, R(x,y)=0. To test the threshold effect, we applied different *T* values in the analysis. Figure 5c shows the effect of the threshold on the statistic distribution of “*R(x,y)*”, where S0, S1, S2 and S3 denote the T values as the average plus 0, 1, 2 and 3 times standard deviation of SIA values of pixels. Figure 5d show the resultant R(x,y) images using S1 for different concentrations. The signal-to-background ratio is substantially improved for low-concentration images, while high-concentration images remain the same. Such a threshold method can help enhance the detection limits for low concentrations due to the effective elimination of background noise.

**Figure 6:**
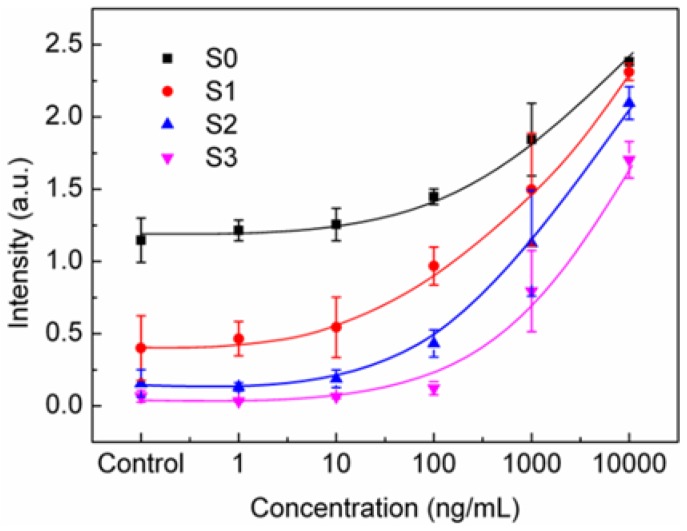
The mean value of SPR responses for different anti-BSA concentrations and threshold values. S0, S1, S2, and S3 correspond to the average plus 0, 1, 2, 3 times standard deviations of the control experiments, respectively (*n* = 3).

Figure 6 shows mean value M of R(x,y) and standard deviation for different concentrations and threshold values; we plot the deviations for the measurements in different SPR chips. The control signal was measured in DI water without dissolving any anti-BSA. In this plot, the standard deviation was obtained from the results of different SPR chips. Due to the fabrication errors between chips, the SPR response had a large deviation for different chips. It is noted that the threshold value plays an important role in the image analysis. For larger T values (S2 and S3), the threshold is too large to remove the real SPR signals. As a result, the SPR signals are reduced for high concentrations and close to zero for low concentrations. The optimal threshold value is around one standard deviation of the control experiments. In this case, undesired noises are efficiently eliminated, also substantially improving the dynamic range, as shown in Figure 6. The signal range from 10 ng/mL to 10 μg/mL is increased by about 1.6 times. Considering only the stability of the measurement system, the LOD of anti-BSA, defined by 3 × STD, was 2.13 ng/mL for S0 and reduced to 1.23 ng/mL for S1. However, when considering the large variation of SPR chips, the LOD was increased. Nevertheless, using the threshold method, the LOD can also be improved as compared to the simple SIA process. In Figure 6, the LODs for S0, S1 and S2 were 461, 279, and 110 ng/mL, respectively. This shows improved LOD using our proposed method.

### 3.3. Experiments in Phosphate-Buffered Saline (PBS)

**Figure 7:**
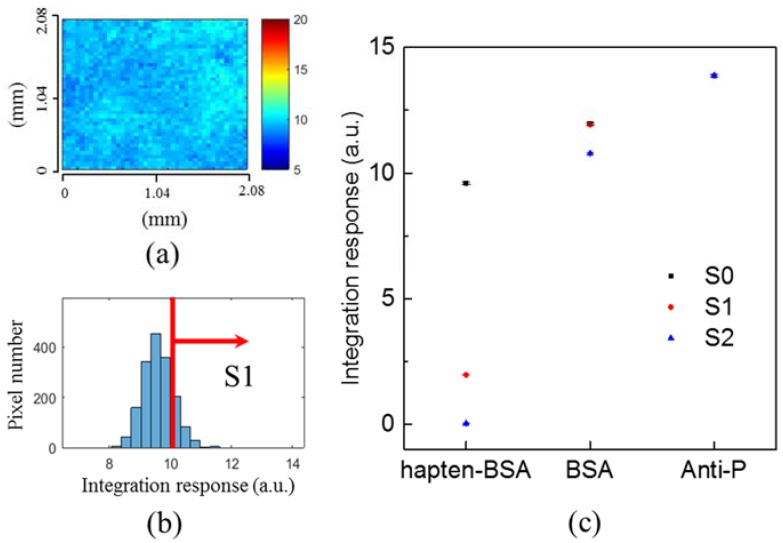
(**a**) SPR image of the hapten-BSA. (**b**) The histogram of the SPR image pixels. The red line shows the threshold value for the analysis. (**c**) The processed signals for S0 (without threshold process), S1 (with one threshold value) and S2 (with twice threshold values) for hapten-BSA, BSA blocking and anti-P images. The error bar was obtained from three measurements.

In this work, the biosensing is shown using BSA and anti-BSA immunoassay in DI water. DI water may cause aggregation formation problems. In order to efficiently dissolve proteins and make sure that the antibody–antigen binding occurs effectively, phosphate buffered silane (PBS) was also tested. In the experiment, the probe was hapten-bovine serum albumin (hapten-BSA) dissolved in PBS with a concentration of 100 μg/mL. It was dropped into the well for 1 h. A layer of hapten-BSA was immobilized on the chip surface due to the covalent binding between the amino group of BSA and the gold surface. The unbound hatpen-BSA was washed away by the PBS buffer. The chip surface was further blocked by coating 1% BSA for 1 h. After PBS washing, 100-μg/mL anti-P was dropped for 1 h for the interaction with hapten-BSA. The biolayer thickness on the chip surface was sequentially increased from the hapten-BSA, BSA blocking to anti-P. Figure 7a shows the SPR image of the hapten-BSA. Figure 7b shows the histogram of the SPR image pixels. The red line shows the threshold value for the analysis. Figure 7c shows the processed signals for S0 (without threshold process), S1 (with one threshold value), and S2 (with twice threshold values) for hapten-BSA, BSA blocking and anti-P images. The SPR signals increased with the biolayer thickness. As compared to S0, the threshold method shows great improvement on the changes of signals for thin biolayer thickness, which is consistent with the case of low-concentration antibody as shown in Figure 6.

## 4. Conclusions

We fabricated multiple-well SPR fluidic chips for high-throughput, label-free biomolecular detection. By contrast with conventional spectral measurements for each well, a spectral scanning image method was used for recording the SPR spectral images of each cell. The SPR chips were fabricated using nanoimprinting technology on plastic substrates. The fabrication was simple and cheap. The SPR was generated by the periodic gold-capped nanowire arrays. It forms a sharp resonance due to the Fano coupling effect. In the process of SPR signals, we applied two analytic approaches for both spectral and spatial information. One was the SIA which took a whole consideration of the peak wavelength shift and SPR intensity change. The SIA method can enhance the signals by about two orders of magnitude as compared to the wavelength shift method. The other was the threshold method in the image process. It removed the background noises. With one standard deviation of pixel values as a threshold, the signal-to-background ratios for low-concentration samples can be greatly increased. The LOD of the antibody can be improved by 1.73 times from 2.13 ng/mL to 1.23 ng/mL. Considering the antibody weight, which is about 150 kDa, the detection limit can be decreased to 8.2 pM. Using the spectral scanning image system and analysis by the SIA and threshold method, multiple kinetic responses with spectral and spatial information on different sensing areas can be measured.

## Supplementary Materials

The following are available online at http://www.mdpi.com/1424-8220/18/7/2181/s1, Video S1: Colormaps of R(x,y) distribution for *t* = 50–150 min displaying in sequence.
